# Abundant expression of TIM-3, LAG-3, PD-1 and PD-L1 as immunotherapy checkpoint targets in effusions of mesothelioma patients

**DOI:** 10.18632/oncotarget.21113

**Published:** 2017-09-21

**Authors:** Elly Marcq, Jorrit De Waele, Jonas Van Audenaerde, Eva Lion, Eva Santermans, Niel Hens, Patrick Pauwels, Jan P. van Meerbeeck, Evelien L.J. Smits

**Affiliations:** ^1^ Center for Oncological Research, University of Antwerp, Antwerp, Belgium; ^2^ Laboratory of Experimental Hematology, University of Antwerp, Antwerp, Belgium; ^3^ Interuniversity Institute for Biostatistics and Statistical Bioinformatics, Hasselt University, Diepenbeek, Belgium; ^4^ Center for Health Economics Research and Modelling Infectious Diseases, University of Antwerp, Antwerp, Belgium; ^5^ Department of Pathology, Antwerp University Hospital, Antwerp, Belgium; ^6^ Thoracic Oncology/MOCA, Antwerp University Hospital, Antwerp, Belgium

**Keywords:** mesothelioma, immune checkpoints, effusions, tumor microenvironment, flow cytometry

## Abstract

Malignant pleural mesothelioma (MPM) is an aggressive cancer with an increasing incidence, poor prognosis and limited effective treatment options. Hence, new treatment strategies are warranted which include immune checkpoint blockade approaches with encouraging preliminary data. Research on the immunological aspects of the easily accessible mesothelioma microenvironment could identify prognostic and/or predictive biomarkers and provide useful insights for developing effective immunotherapy.

In this context, we investigated the immune cell composition of effusions (pleural and ascites fluids) from 11 different chemotherapy-treated MPM patients. We used multicolor flow cytometry to describe different subsets of immune cells and their expression of immune checkpoint molecules TIM-3, LAG-3, PD-1 and PD-L1. We demonstrate a patient-dependent inter- and intraspecific variation comparing pleural and ascites fluids in immune cell composition and immune checkpoint expression. We found CD4^+^ and CD8^+^ T cells, B cells, macrophages, natural killer cells, dendritic cells and tumor cells in the fluids. To the best of our knowledge, we are the first to report TIM-3 and LAG-3 expression and we confirm PD-1 and PD-L1 expression on different MPM effusion-resident immune cells. Moreover, we identified two MPM effusion-related factors with clinical value: CD4+ T cells were significantly correlated with better response to chemotherapy, while the percentage of PD-L1^+^ podoplanin (PDPN)^+^ tumor cells is a significant prognostic factor for worse outcome. Our data provide a basis for more elaborate research on MPM effusion material in the context of treatment follow-up and prognostic biomarkers and the development of immune checkpoint-targeted immunotherapy.

## INTRODUCTION

Malignant pleural mesothelioma (MPM) is a highly aggressive and fatal cancer that is most commonly associated with asbestos exposure [1]. Due to differences in asbestos contact, MPM incidence varies not only between but also within countries [2]. Although it used to be a rare disease, its incidence has been increasing in recent years and it is expected to continue in the next decades. This is mainly due to an ongoing asbestos use in developing countries, as well as the long latency period between asbestos exposure and the development of disease [3]. MPM is characterized by a bad prognosis. Palliative platinum-antifolate chemotherapy has a significant but limited impact on patients’ outcome with a median overall survival of about one year [4, 5]. Based on this poor prognosis and the increasing incidence, novel therapeutic strategies for MPM are urgently required.

Preliminary clinical evidence suggests a critical role for the immune system in protection against MPM [6–8] and recent promising clinical results have been reported on inhibition of the immune checkpoints cytotoxic T-lymphocyte antigen-4 (CTLA-4) and programmed death-1 (PD-1) or its ligand PD-L1 in MPM [9–14]. It is postulated that blocking immune checkpoints reactivates silenced immune responses by preventing immune cell exhaustion and tolerance. Gaining more insight in the immunological aspect of the tumor microenvironment (TME) is of great interest in order to identify biomarkers and to unravel these silenced immune responses in order to identify possible new targets for MPM treatment. Therefore, we previously investigated the immune composition and the expression of immune checkpoints in human MPM tissue samples [15]. Now, we want to focus on pleural and peritoneal effusions. These effusions are often present in MPM patients [3] and are easily collected via thoracocentesis or paracentesis. Tumor cells and immune cells have been described to be present in pleural effusions [3, 16]. Extensive investigation of the cellular composition in relation to the expression of immune checkpoints could help to identify prognostic markers in the effusions. It could also provide useful information on immunomodulatory molecules that are expressed in the TME potentially dampening the antitumor immune responses, helping forward the development of novel targeted therapies.

The few studies on effusions of mesothelioma patients mainly report on the presence of a limited array of immune cells, being CD4^+^ and CD8^+^ T cells and macrophages [17–20]. One of those studies also describes the expression of PD-1 and PD-L1 [18], but to the best of our knowledge nothing has been described about the expression of T cell immunoglobulin mucin-3 (TIM-3) and lymphocyte activation gene-3 (LAG-3) in mesothelioma effusions. The latter two are upcoming immune checkpoints that are gaining more attention due to interesting preclinical results in different tumor types [21–25] and ongoing clinical trials testing specific blocking antibodies (NCT01968109; NCT02817633). LAG-3 is expressed on the surface of activated T cells. It also binds to MHCII molecules expressed on antigen presenting cells but in contrast to CD4, LAG-3 exerts a negative regulatory effect on the proliferation and activation of T lymphocytes [21, 22]. Expression of TIM-3 and its ligand galectin-9 has been described on several immune cells and galectin-9 can also be overexpressed by cancer cells [26, 27]. Interaction of TIM-3 with galectin-9 results in decreased immune cell functioning and even immune cell death. We have recently described the absence of LAG-3 and the presence of TIM-3 on tumor cells and stromal immune cells in mesothelioma tissue [15].

In this study, we characterized the cellular composition and TIM-3, LAG-3, PD-1 and PD-L1 immune checkpoint expression of pleural and ascites fluid samples collected from MPM patients at least one month after chemotherapy treatment. Relating to patients’ outcome and response to chemotherapy, we identified two effusion-related factors with clinical value.

## RESULTS

### Immune cell composition of MPM effusions is variable and patient-dependent

Flow cytometry was used to analyze the immune cell composition in five ascites fluids and six pleural fluids collected from MPM patients at least one month after chemotherapy treatment. All cell types analyzed could be detected in the fluids with a variable distribution (Table 1). Predominant cells were CD3^+^CD4^+^ T cells, CD64^+^ macrophages and CD11c^+^ or CD303^+^ dendritic cells (DCs) with percentages up to 51.7%, 38.8% and 43.4% of viable cells, respectively. CD3^+^CD8^+^ T cells, CD3^-^CD56^+^ natural killer (NK) cells and CD19^+^ B cells were less present, with maxima of 16.9%, 13.5% and 2.9% viable cells per sample. Based on the expression of podoplanin (PDPN), a marker for MPM tumor cells [28–31], ascites fluids contained slightly more PDPN^+^ tumor cells compared to pleural fluids (median 0.5% vs 0.1%). Higher percentages of immune cells were noted in the ascites fluids compared to the pleural fluid samples, with *p*-values ranging from 0.247 to 0.930 (Table 1).

**Table 1:** Immune composition of pleural and ascites fluid samples from MPM patients

	CD4+ T CELLS (%)	CD8+ T CELLS (%)	NK CELLS (%)	DCs (%)	B CELLS (%)	MACROPHAGES (%)	TUMOR CELLS (%)
*Pleural fluid*							
Sample 1 (E)	2.98	1.23	1.10	36.97	0.30	17.00	0.58
Sample 2 (S)	2.99	0.80	0.04	0.59	1.21	0.46	0.02
Sample 3 (S)	31.16	5.84	0.83	4.09	1.77	3.31	0.04
Sample 4 (E)	4.45	0.91	0.09	0.67	0.40	0.46	0.02
Sample 5 (E)	33.83	2.45	1.27	16.77	2.86	15.70	0.11
Sample 6 (E)	29.41	7.71	2.41	27.33	1.72	26.03	0.26
MEDIAN	16.93	1.84	0.96	10.43	1.47	9.50	0.08
SD	15.41	2.92	0.88	15.28	0.96	10.62	0.22
RANGE_MIN_	2.99	0.80	0.04	0.67	0.30	0.46	0.02
RANGE_MAX_	33.83	7.71	2.41	36.97	2.86	26.03	0.60
*Ascites fluid*							
Sample 1 (E)	20.47	2.78	2.35	12.26	1.27	11.20	0.53
Sample 2 (E)	4.15	1.95	0.35	43.43	0.43	38.80	12.86
Sample 3 (E)	51.75	11.83	0.85	12.60	2.07	11.87	0.54
Sample 4 (E)	18.12	16.88	13.48	28.17	0.78	24.57	0.86
Sample 5 (N)	0.80	0.79	0.05	0.06	0.05	0.06	0.01
MEDIAN	18.12	2.78	0.85	12.60	0.78	11.87	0.54
SD	20.17	7.12	5.69	16.78	0.78	14.83	5.54
RANGE_MIN_	0.80	0.79	0.35	0.06	0.05	0.06	0.01
RANGE_MAX_	51.75	16.88	13.48	43.43	2.07	38.80	12.86

All percentages are calculated within the total population of viable cells. E, epitheloid; S, sarcomatoid; N, no data.

### Inter and intra sample type variation of PD-1, TIM-3 and LAG-3 expression

**Figure 1:**
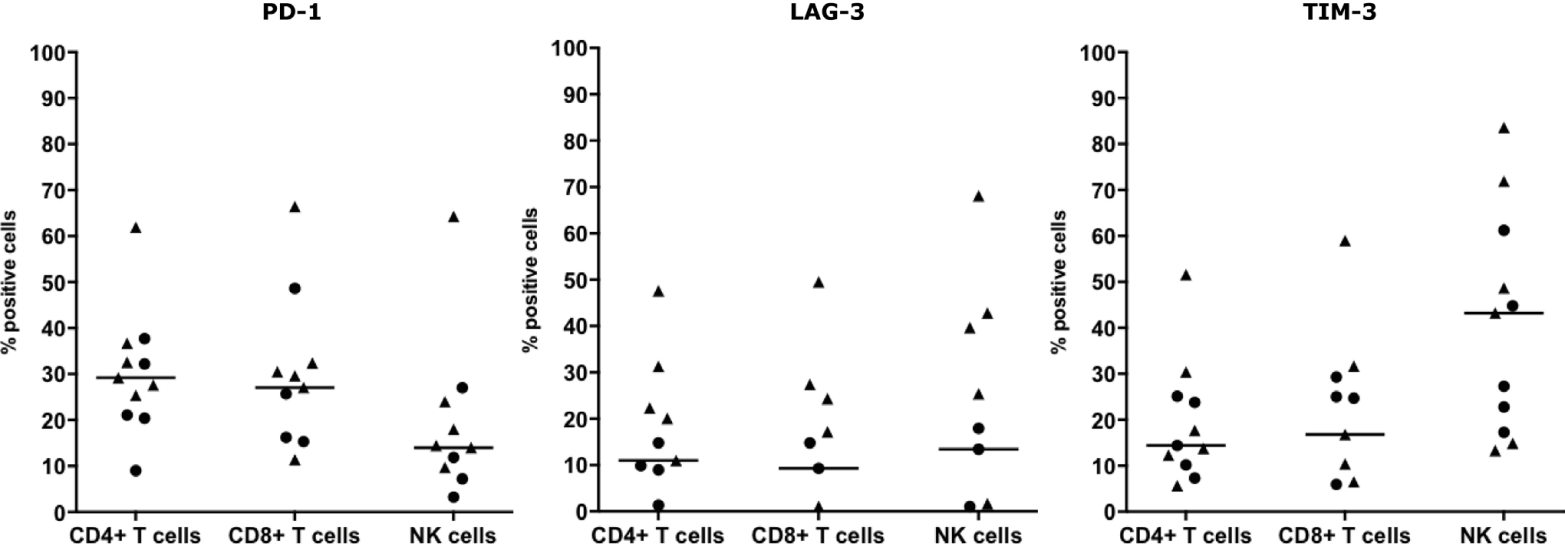
T cell and NK cell immune checkpoint surface expression in MPM pleural and ascites fluids Surface expression of PD-1, LAG-3 and TIM-3 on CD3^+^CD4^+^ T cells, CD3^+^CD8^+^ T cells and CD3^-^CD56^+^ NK cells from five ascites (·) and six pleural (▲) fluids. Percentages of marker-positive cells were determined using the overton subtraction tool in the FlowJo software. The bar represents the median value within all samples.

The immune checkpoints PD-1, LAG-3 and TIM-3 were expressed on CD3^+^CD4^+^ T cells, CD3^+^CD8^+^ T cells and on NK cells in both fluid types. PD-1 expression was found in all samples on CD8^+^ T cells and in ten samples on CD4^+^ T cells and/or NK cells. Positivity of LAG-3 was seen in nine samples on CD4^+^ T cells, in seven samples on CD8^+^ T cells and in eight samples on NK cells. Expression of TIM-3 was observed in all samples on CD4^+^ T cells and NK cells and in nine samples on CD8^+^ T cells (Figure 1). Figure 1 and Table 2 depict the distributions and proportions of PD-1, LAG-3 and TIM-3 expression on CD3^+^CD4^+^ and CD3^+^CD8^+^ T cells and NK cells. All pleural samples were positive for PD-1 on all three cell types, whereas PD-1 was expressed on CD8^+^ T cells and NK cells in four of the five ascites samples. PD-1 positivity varied in each cell type, ranging from 9% to 61.9% for the CD4^+^ T cells, 11.4% to 66.4% for the CD8^+^ T cells and 3.28% to 64.3 % for the NK cells. LAG-3^+^ cells were detected in a higher proportion of pleural fluids compared to ascites fluids. LAG-3 expression on a per cell-base, is highest on NK cells in both fluid types. A wide range of LAG-3 expression per cell type was recorded, from 1.3–47.6% LAG-3^+^ CD4^+^ T cells, 1.1–49.5% LAG-3^+^ CD8^+^ T cells and 1.0–68.1% LAG-3^+^ NK cells (Table 2). TIM-3 was expressed on most of the NK cells while less CD3^+^CD4^+^ and CD3^+^CD8^+^ T cells were positive (Figure 1). Based on the difference in mean fluorescence intensity (ΔMFI) values, the highest expression per cell was seen on NK cells. Like PD-1 and LAG-3, the proportion of TIM-3^+^ positive cells varied strongly, illustrated by 5.7–51.6% TIM-3^+^CD4^+^ T cells, 5.9–59.7% TIM-3^+^CD8^+^ T cells and 13.3–83.6% TIM-3^+^ NK cells (Figure 1).

**Table 2:** Immune checkpoint expression of MPM effusion-resident immune cells

		CD4+ T CELLS	CD8+ T CELLS	NK CELLS
*Pleural fluids (n = 6)*				
PD-1	Positive samples (%)	6/6	6/6	6/6
	ΔMFI range_MIN-MAX_	772–4902	1032–7066	763–3932
LAG-3	Positive samples (%)	5/6	5/6	5/6
	ΔMFI range_MIN-MAX_	61–1221	60–7311	231–14819
TIM-3	Positive samples (%)	6/6	5/6	6/6
	ΔMFI range_MIN-MAX_	91–1539	1225–5996	1297–12541
*Ascites fluids (n = 5)*				
PD-1	Positive samples (%)	5/5	4/5	4/5
	ΔMFI range_MIN-MAX_	689– 4981	783–6009	215–4708
LAG-3	Positive samples (%)	3/5	2/5	3/5
	ΔMFI range_MIN-MAX_	154–867	208–230	36–1410
TIM-3	Positive samples (%)	5/5	4/5	5/5
	ΔMFI range_MIN-MAX_	368–5194	338–6706	3036–9595

Variation in the intensity of expression is shown by the range of Δ MFI values. MFI, Mean Fluorescence Intensity.

### PD-1 ligand-1 expression is highest on PDPN^+^ tumor cells

**Figure 2:**
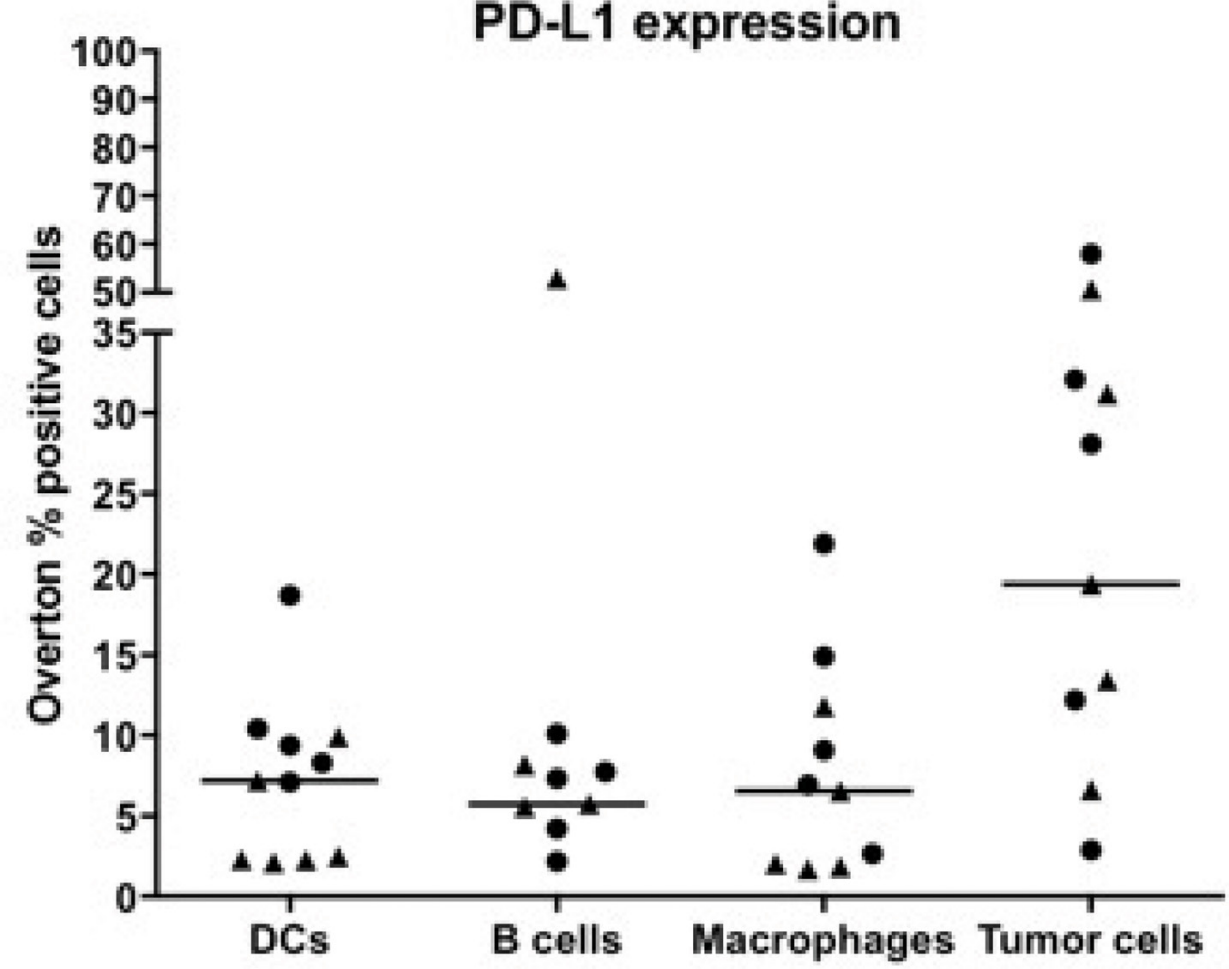
Expression of PD-L1 on immune cells and tumor cells in pleural and ascites fluids of MPM patients Data of eleven different patient samples are shown: five ascites (·) and six pleural (**▲**) fluids. Percentages of marker-positive cells were determined using the overton subtraction tool in the FlowJo software. The bar represents the median value within all samples.

PD-1 ligand-1, PD-L1, expression was detected on DCs, B cells, macrophages and PDPN^+^ MPM tumor cells in all ascites samples, while in pleural samples PD-L1 expression varied per cell type (Figure 2, Table 3). Positivity of PD-L1 on DCs was found in all samples, while positivity of PD-L1 on B cells was found in nine samples and ten samples showed expression of PD-L1 on macrophages and PDPN^+^ tumor cells. Highest PD-L1 expression was found on the PDPN^+^ tumor cells in both fluid types, with a median expression of 19.4% PD-L1^+^ cells and a range of 20 up to 14524 on a per cell basis (Table 3). Similar to the immune checkpoints, there is a broad range of PD-L1 positivity between and within the fluid types. In ascites fluid samples, more PD-L1^+^ DCs (*p* = 0.052) and PD-L1^+^ macrophages (*p* = 0.052) were present compared to the pleural fluid samples.

**Table 3:** Overview of PD-L1 expression on immune cells and MPM tumor cells in eleven different MPM fluid samples

		DCs	B CELLS	MACROPHAGES	TUMOR CELLS
*Pleural fluid*					
PD-L1	Positive samples (%)	6/6	4/6	5/6	5/6
	ΔMFI range_MIN-MAX_	305–749	9–1684	128–972	20–14524
*Ascites fluid*					
PD-L1	Positive samples (%)	5/5	5/5	5/5	5/5
	ΔMFI range_MIN-MAX_	159–1296	10–477	331–1801	955–2679

Variation in the intensity of expression is shown by the range of ΔMFI values. MFI, Mean Fluorescence Intensity.

### Expression of immune checkpoints is correlated with immune cell activation

**Figure 3:**
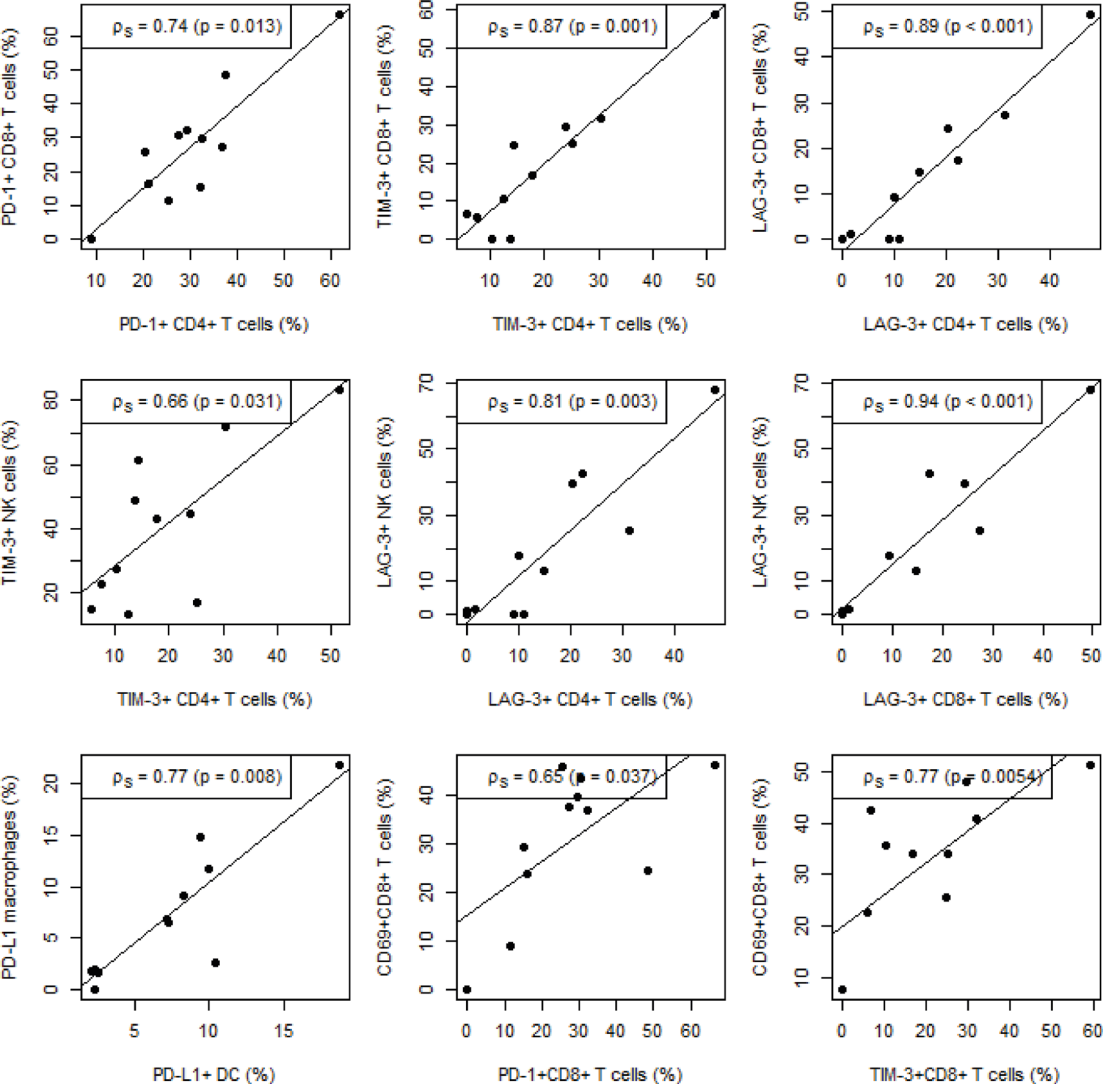
Correlation between immune checkpoint expression on different immune cells and immune cell activation Significant correlations between immune checkpoint expression on different subsets of immune cells as well as immune cell activation are depicted on the scatter plots. Spearman correlation (ρ_s_) coefficients and corresponding *p*-values are shown for each correlation. The lines represent linear regression fits.

We investigated correlations between immune cell types expressing immune checkpoints. Expression of PD-1, TIM-3 and LAG-3 on CD4^+^ T cells was correlated with their expression on CD8^+^ T cells [spearman correlation coefficient (ρ_s_) = 0.74, *p* = 0.013; ρ_s_ = 0.87, *p <* 0.001; ρ_s_ = 0.89, *p <* 0.001, respectively; Figure 3]. TIM-3 and LAG-3 expression was also correlated with CD4^+^ T cells and NK cells (ρ_s_ = 0.66, *p* = 0.031; ρ_s_ = 0.81, *p* = 0.003, respectively). For LAG-3 but not TIM-3, expression on CD8^+^ T cells was also strongly correlated with its expression on NK cells (ρ_s_ = 0.94, *p <* 0.001). For the PD-1 ligand PD-L1, a positive correlation was found for its expression on DCs and macrophages (ρ_s_ = 0.77, *p* = 0.008, Figure 3). PD-1, LAG-3 and TIM-3 expression was also put in relation with an activated phenotype of CD3^+^CD4^+^, CD3^+^CD8^+^ T cells and CD3-CD56^+^ NK cells, assessed by CD69 expression. CD69 expression was significantly correlated with the expression of PD-1 and TIM-3 on CD3^+^CD8^+^ T cells (ρ_s_ = 0.65, *p* = 0.037; ρ_s_ = 0.77, *p* = 0.005; Figure 3).

### Patient-matched MPM tissue and effusion samples show specific cell and immune checkpoint expression

Matched tissue and effusion samples from four patients allowed us to compare their immune cell composition and immune checkpoint expression. Immunohistochemistry on paraffin-embedded diagnostic tissue samples [15] and multi-parameter flow cytometry on pleural fluids (*vide* Materials and methods) was assessed for the presence of CD4^+^ and CD8^+^ T cells and macrophages and the expression of PD-L1 on tumor cells and PD-L1, LAG-3 and TIM-3 on lymphocytes. Patient-matched comparison indicates that the immune composition and immune checkpoint expression differs between the sample types (Supplementary Table 1). Higher percentages of macrophages and lymphocytes were noted in the tissue samples compared to the fluid samples. We also observed an inter sample type variation in immune checkpoint expression on lymphocytes and PD-L1 expression on tumor cells present in the fluid and tissue samples.

### Two MPM effusion-related cellular parameters with clinical value

**Figure 4:**
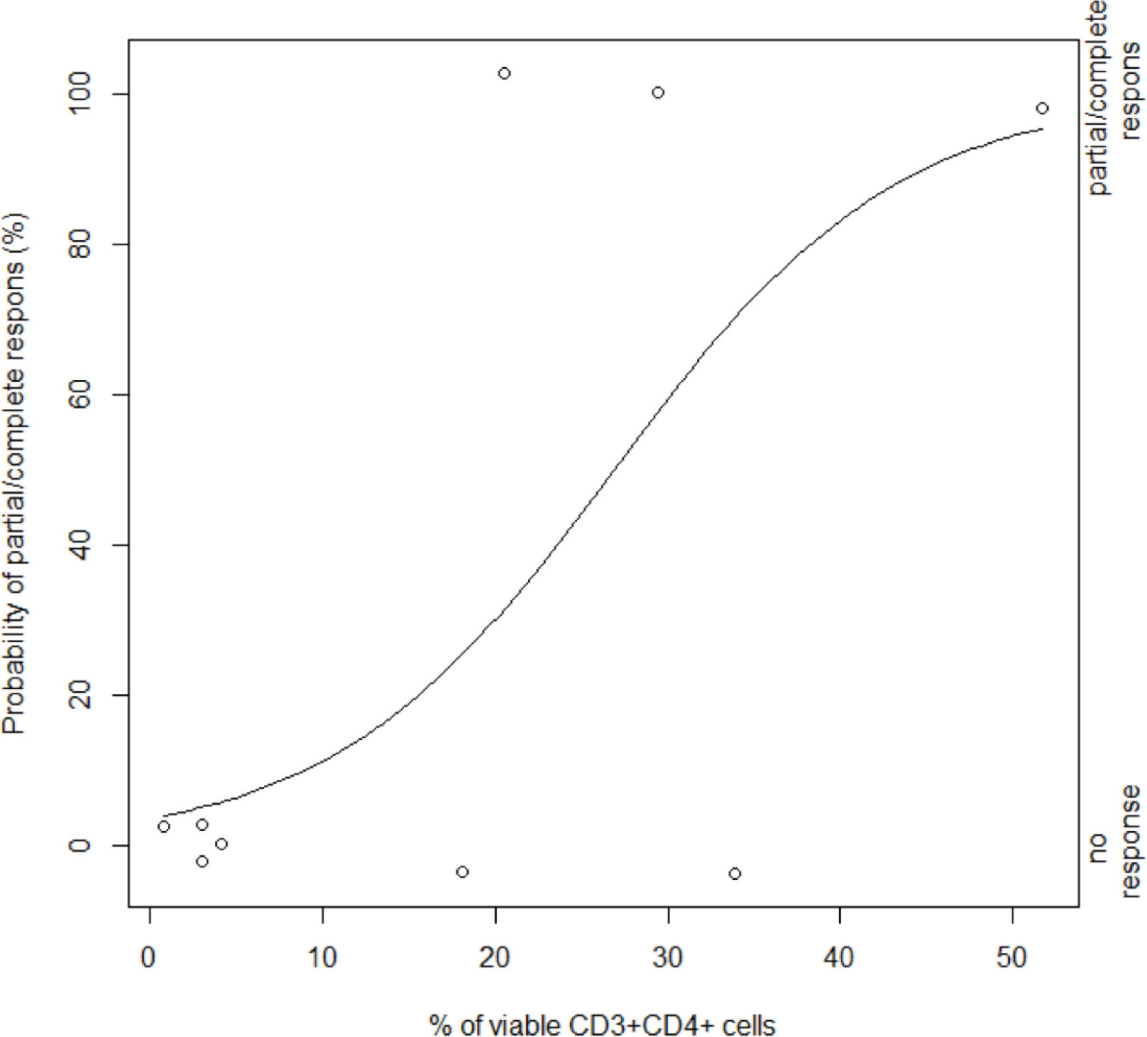
Dependency between chemotherapy response and the presence of CD3+CD4+ T cells after treatment Plot of response on chemotherapy (y-axis) versus percentage of viable CD3+CD4+ cells in the fluids after treatment (x-axis). Observations within our cohort are represented by the empty dots (right y-axis). The curve depicts the estimated probability of partial/complete response based on a univariate logistic regression model (*p* = 0.033; left y-axis).

**Figure 5:**
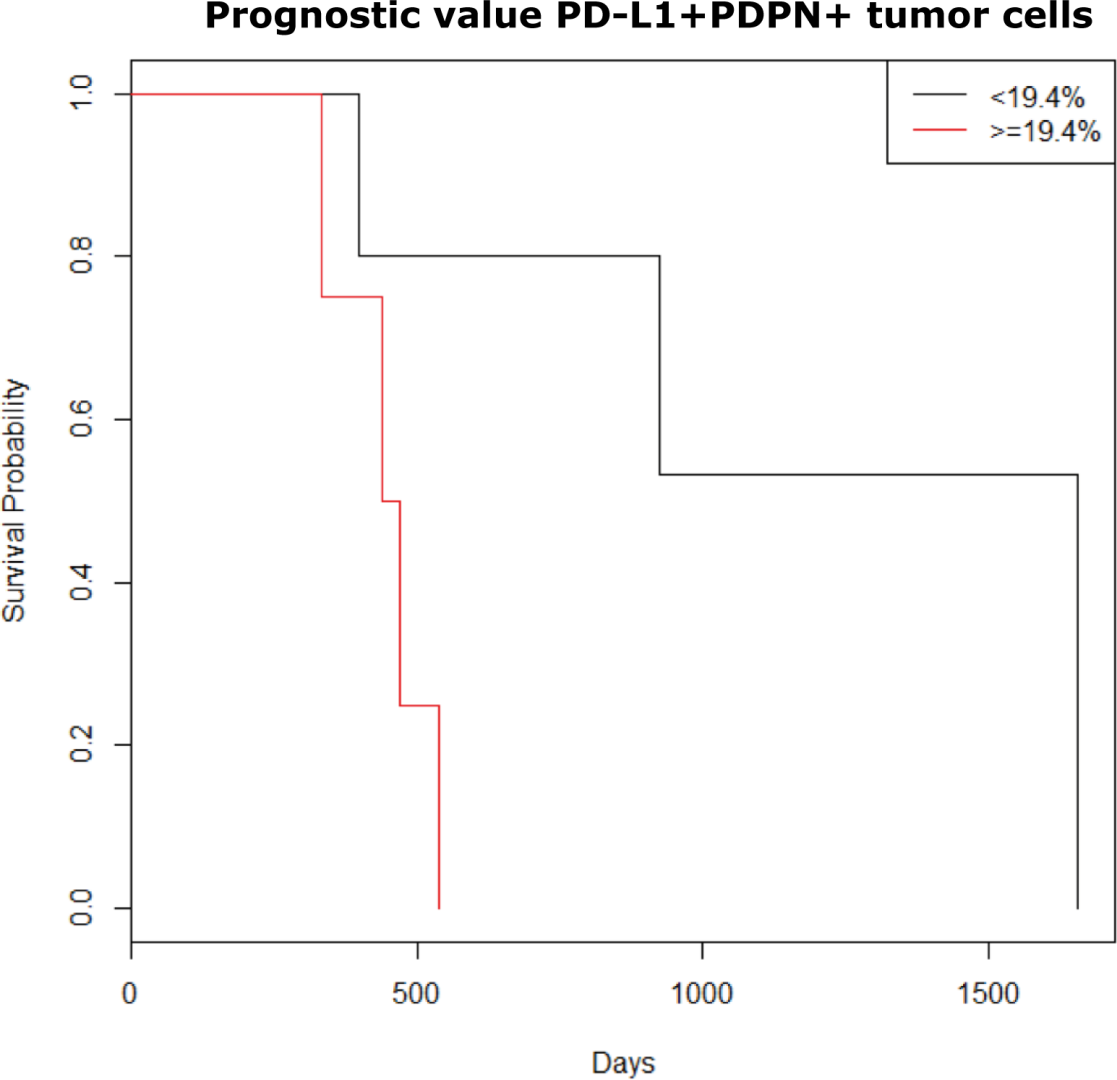
Kaplan Meier overall survival according to percentages of PD-L1+ tumor cells present in MPM fluid samples The variable PD-L1+PDPN+ tumor cells (%) was divided into two groups based on the median value for the percentage of PD-L1+PDPN+ cells in our samples (median = 19.4%). Univariate analysis showed prognostic significance for PD-L1+PDPN+ tumor cells (*p* = 0.012). After multivariate adjustments, it remained an independent negative prognostic factor.

Searching for parameters with a potent clinical value, univariate analysis indicates that there is a significant correlation between CD3^+^CD4^+^ T cells in MPM effusions after chemotherapy [Mc Fadden's pseudo coefficient of determination (R) = 0.63, *p* = 0.033]. More specifically, partial/complete response to cisplatin/pemetrexed-based chemotherapy was more often noted in patients with high percentages of CD4+ T cells [odds ratio (OR) = 1.13; Figure 4]. Small sample size and the use of discrete variables (0 or 1) made multivariate analysis not feasible. Relating with clinical outcome, PD-L1 expression on effusion-resident PDPN^+^ tumor cells has significant impact on the patient's survival [risk ratio (RR) = 1.10, *p* = 0.011; Figure 5]. The likelihood ratio obtained after Cox regression analysis shows that a 1% increase of PD-L1^+^ PDPN^+^ tumor cells will increase the risk of dying with a factor of 1.10.

## DISCUSSION

Our study shows different subsets of immune cells to be present in MPM pleural and ascites fluid samples. Although statistically not significant, we did observe differences regarding the immune constitution between both fluid types as well as within the same fluid type. This suggests that the immune constitution of MPM fluid samples depends on the patient rather than on the fluid type but more evidence is needed. The different subsets of immune cells showed distinct expression profiles for several immune checkpoints and PD-L1. Like the immune composition, also the expression profiles varied within the different samples as well as between the two different sample types.

Our results confirm the study from Khanna et al. [18] that describes the presence of PD-L1^+^ tumor cells and both PD-1^+^ and PD-L1^+^ infiltrating immune cells in effusions of mesothelioma patients. The fraction of T cells expressing PD-1 varied a lot in this study (12% to 83%) which is in line with the variation seen in our own samples with a range from 9% up to 67% PD-1^+^ T-cells. They also reported basal PD-L1 expression in all their mesothelioma effusion samples with a broad range for the PD-L1 ΔMFI values, which is again in concordance with our own data.

While PD-L1 expression in MPM tissue samples has already been reported to be associated with decreased overall survival [32, 33], as far as we know we are the first to report this for MPM fluid samples. Based on flow cytometric PDPN staining we found only few circulating PDPN^+^ tumor cells were present in ascites samples and even less were found in pleural fluids. Miyoshi et al. [34] made paraffin-embedded cell blocks from pleural fluids and used them for immunohistochemical staining. They found tumor cells in those cell blocks based on cytology, confirming our data about the presence of PDPN^+^ tumor cells in the fluid samples. Due to the tight organization of the tumor tissue as well as the surrounding mesothelial cells, tumor cells are expected not to float abundantly into patients’ effusions.

Our data show that expression of PD-L1 on PDPN^+^ tumor cells in our fluid samples is a poor prognostic factor. Our study is limited by its small sample size which is why we used non-parametric tests for statistical analyses. The fact that PD-L1 expression on PDPN^+^ tumor cells was found to be a significant factor in our small cohort supports the need for further research in a larger cohort. On top of that, the prognostic value that we found for PD-L1 expression on MPM tumor cells is also supported by other studies in mesothelioma tissue samples [33, 35, 36] as well as in other cancer types [37–39]. Since the tumor cells express PD-L1, they might induce the expression of PD-1 and PD-L1 on surrounding immune cells via activation of the PD-1/PD-L1 pathway [40]. The associated decreased survival can be explained by an overstimulation of the PD-1/PD-L1 pathway leading to immune cell exhaustion with an inefficient antitumor response as final result. It would be interesting to investigate in future studies if the expression of PD-1 and PD-L1 in effusions might be good predictive factors for response to anti PD-1/PD-L1 blocking therapy.

As tissue samples from MPM patients are not always available and most epithelioid mesothelioma patients develop effusions over time [1, 41], fluids might offer an alternative for a repeat biopsy for pharmacodiagnostic or prognostic purposes. Multi-parametric analysis of MPM fluids could then be used as an alternative for more elaborate immunohistochemistry on tissue samples for predictive (when the samples are obtained prior to treatment) and prognostic purposes.

In addition to PD-1 and PD-L1, also the immune checkpoints TIM-3 and LAG-3 can affect the antitumor response by inhibiting lymphocyte activity. Therefore, investigation of immune checkpoint expression as well as immune cell profiling of the TME might be of great value to select patients who are most likely to benefit from chemo- or immunotherapy and to provide additional information on patient's outcome. To our knowledge, we are the first to describe the expression of TIM-3 and LAG-3 on CD4^+^ and CD8^+^ T cells and CD3-CD56^+^ NK cells in effusions of mesothelioma patients. Like PD-1 expression, we observed a considerable interpatient variation regarding the immune cell expression of TIM-3 and LAG-3. NK cells showed the highest expression of both markers, confirming previous MPM-unrelated reports [42–44]. Based on our results from MPM fluids not only TIM-3 could be an interesting target for MPM, but also LAG-3 might offer new opportunities. Clinical trials with TIM-3 and LAG-3 blockade in various solid tumor types are ongoing (NCT02817633; NCT02608268; NCT01968109; NCT03005782). Interestingly, our recent work [15] showed no expression of LAG-3 on tumor cells and immune cells in MPM tissue samples while our data in fluid samples showed its presence on T cells as well as NK cells. Since effusions most often occur in an inflammatory context which is known to influence the expression of immune checkpoints [45] it is quite possible that the fluid samples do not reflect the tumor microenvironment. Comparison of four patient-matched tissue and fluid samples demonstrates that the two milieus have a distinct immune composition and expression of immune checkpoints, which might be an explanation for the discrepancy observed between tissue and fluid samples. From our small sample size, it is suggested that MPM fluid samples do not have the same cellular characteristics as MPM tissue samples, which has also been suggested by Lievense et al. [46]. They compared the presence of T cells and macrophages in pleural effusions of 5 MPM patients with matched tissue samples and suggested that the immune cell composition of the effusions does not necessarily reflect the properties of the tumor tissue. Our results also confirm the presence of CD4^+^ T cells, CD8^+^ T cells and macrophages in pleural effusions of MPM patients [18–20]. Another explanation for the discrepancy between MPM tissue sample and effusion results for LAG-3 might be the use of different antibody clones for immunohistochemistry and flow cytometry. It might be that the expression levels are too low to be detected with immunohistochemistry, while the signal can be picked up by the flow cytometry antibody due to a higher sensitivity.

In general, PD-1, TIM-3 and LAG-3 were expressed on immune cells present in the majority of pleural and ascites samples. Our data showed that expression of an immune checkpoint on CD4^+^ T cells was positively correlated with its expression on CD8^+^ T cells and that the expression of TIM-3 or LAG-3 on CD4^+^ T cells was also correlated with their expression on NK cells. These correlations might be explained by the close interaction between these cell types in order to elicit an antitumor immune response. CD4^+^ T cells provide help to CD8^+^ T cells and NK cells by secreting immunostimulatory cytokines [47–49]. Since expression of PD-1, TIM-3 and LAG-3 has been reported on activated immune cells [26, 50, 51], stimulation of these cells will result in an increased expression on all three cell types. This idea is supported by the positive correlations that we found between CD69 and PD-1 expression and between CD69 and TIM-3 expression on CD8^+^ T cells. In the evaluated patient population of cisplatin/pemetrexed-treated patients who developed effusions over time, we observed that the percentage of CD3^+^CD4^+^ T cells is significantly correlated with the patients’ response to chemotherapy. Since cisplatin has been reported to promote the recruitment and proliferation of immune effector cells [52], this association could be explained by cisplatin-stimulated proliferation of these cells which ultimately could result in a more effective antitumor response.

## MATERIALS AND METHODS

### Patients and samples

The human biological material used in this publication was provided by Biobank@UZA (Antwerp, Belgium; ID: BE71030031000; Belgian Virtual Tumorbank funded by the National Cancer Plan). Five ascites fluid samples and six pleural fluid samples were collected from eleven different MPM patients who received first-line chemotherapy (cisplatin + pemetrexed) at least one month before sample collection. Patients’ clinicopathological parameters are detailed in Table 4. There were no significant differences between the parameters of patients with ascites fluids and the ones with pleural fluids. Matched tissue samples were available from four patients and were used for immunohistochemical analysis as described previously [15]. This study has been approved by the Ethics Committee of the Antwerp University Hospital/University of Antwerp (EC 14/39/397).

**Table 4:** Clinicopathological parameters of the MPM patient population

CHARACTERISTICS	PLEURAL FLUIDS (*n*, %)	ASCITES FLUIDS (*n*, %)	*P*-VALUE
*Number of samples (N)*			
	6	5	
*Age (years)*			0.170
Median	66	61	
Range	60–73	41-67	
*Sex*			0.354
Male	6 (100%)	3 (60%)	
Female	0	2 (40%)	
*Histological subtype*			0.240
Epitheloid	3 (50%)	4 (80%)	
Sarcomatoid	2 (33%)	0	
No data	1 (17%)	1 (20%)	
*Smoker*			1.000
No	4 (67%)	1 (20%)	
Yes	2 (33%)	3 (60%)	
No data	0	1 (20%)	
*Professional asbestos exposure*			/
No	0	0	
Yes	5 (83%)	3 (60%)	
No data	1 (17%)	2 (40%)	
*Survival*			0.097
Alive	4 (67%)	0	
Dead	2 (33%)	5 (100%)	
*Laterality*			1.000
Left	2 (33%)	2 (40%)	
Right	4 (67%)	3 (60%)	
*Stage*			0.168
I–II	3 (50%)	0	
III–IV	2 (33%)	5 (100%)	
No data	1 (17%)	0	
*Hemoglobin (g/dL)*			1.000
< 14.6 (low)	4 (67%)	4 (80%)	
≥ 14.6 (high)	2 (33%)	1 (20%)	
*White blood cell count (*×*10*^3^ *cells/ μL)*			/
< 15.5 (low)	6 (100%)	5 (100%)	
≥ 15.5 (high)	0	0	
*Platelet count*			1.000
< 400 (low)	4 (67%)	4 (80%)	
≥ 400 (high)	2 (33%)	1 (20%)	
*Neutrophil/lymphocyte ratio*			0.662
< median^♦^	3 (50%)	2 (40%)	
≥ median^♦^	3 (50%)	3 (60%)	

^♦^median of pleural fluids = 34.5; median of ascites fluids = 62.0.

### Sample collection

Fluid samples, obtained from patients as a standard procedure for symptomatic relief, were collected via paracentesis (ascites) or thoracocentesis (pleura) in sterile tubes and filtered through a cell strainer before centrifugation. A Ficoll-Paque Plus^TM^ (GE Healthcare Life Sciences, Belgium) density gradient centrifugation at 2100 rpm for 20 minutes was performed to collect the immune cells (peripheral blood mononuclear cells) and remove the red blood cells. After aspirating the upper layer, the layer with immune cells was transferred to a new sterile FACS tube diluted with phosphate buffered saline (PBS) and centrifuged at 1500 rpm for 5 minutes. Cell pellets were suspended in fetal bovine serum (FBS) supplemented with 10% dimethylsulfoxide (DMSO) and stored at liquid nitrogen until use.

### Flow cytometry

Frozen samples were thawed in RPMI 1640 medium supplemented with 10% FBS and 0,5% sodiumpyruvate in a 37°C warm water bath. Cells were counted on an ABX Micros 60 automatic cell counter (Horiba Medical, California, USA). Samples were analyzed for the presence of CD3^+^CD4^+^ T cells, CD3^+^CD8^+^ T cells, CD3^-^CD56^+^ NK cells, CD19^+^ B cells, CD64^+^ macrophages, CD11c^+^CD33^+^ DCs and PDPN^+^ tumor cells and for PD-1, PD-L1, TIM-3 and LAG-3 immune checkpoint expression using multicolor flow cytometry. In a first panel, combinations of PD-1-PE (clone MIH4) / LAG-3-PE (clone T47530)/ TIM-3-PE (clone 7D3), CD3-BV510, CD4-APC-H7, CD8-PB, CD69-APC and CD56-FITC (clone B159) conjugated monoclonal antibodies (mAbs) were used. The live/dead^Ò^ red fixable cell stain was added to assess cell viability (Supplementary Figure 1). In a second panel, combinations of PD-L1-PE (clone MIH1), CD19-BV421(clone HIB19), CD64-FITC (clone 10.1), CD11c-PeCy7 (clone B-ly6) and CD303-PeCy7 (clone 201A), CD64-FITC and podoplanin-APC (PDPN, clone NZ-1.3) conjugated mAbs were used. A live/dead aqua fixable cell stain was added to assess cell viability (Supplementary Figure 2). All the antibodies were purchased from Becton Dickinson (BD Biosciencesâ), except for the live/dead stains and the CD8 mAb (Life Technologies^TM^), the CD303 mAb (Biolegendâ, California, USA) and the PDPN mAb (eBioscienceâ, Vienna, Austria). Corresponding species- and isotype-matched antibodies were used as controls. In brief, surface staining with mAbs and live/dead stain was performed for 15 minutes at room temperature, cells were washed (5 min, 1500 rpm) and resuspended in FACS buffer (sheath + 0.1% bovine serum albumine + 0.05% sodiumazide). Samples were acquired with FACSDiva software on a FACSAria II Beckton Dickinson flow cytometer (BD Biosciencesâ). Data were analyzed using FlowJo software (TreeStar inc., Ashland, USA). Results are expressed as ΔMFI (calculated by subtracting MFI values of isotype controls from marker MFI values) and as percentages of marker-positive cells (determined by Overton subtraction of isotype control histograms from marker histograms).

### Statistics

Spearman correlation coefficients were calculated to investigate the correlation between: (i) the expression of immune checkpoints and immune cell markers among the different fluid samples; (ii) the expression of immune checkpoints and immune cell markers in tissue samples and their corresponding fluid sample (*n* = 4); (iii) the expression of immune checkpoints and the activation marker CD69 on different subsets of effusion-resident immune cells. To investigate differences between ascites and pleural fluids, Wilcoxon-rank sum and Fisher exact tests were performed. Patients overall survival was assessed from the date of diagnosis to the date of sample analysis or the date of death. The influence of immunological and clinicopathological parameters on survival was assessed using Cox proportional hazards models. The proportional hazards assumption was tested and could not be rejected for any of the parameters under consideration. Response to chemotherapy was defined using the Response Evaluation Criteria in Solid Tumors (RECIST) [53]. The associations of the subsets of immune cells present in the fluid samples with response to chemotherapy were determined using logistic regression. Variable selection was performed by assessing significance on the 10% level in univariate analyses (*p <* 0.1). Backward model building was used in the multivariate models for which *p*-values < 0.05 were considered statistically significant.

## CONCLUSIONS

With this study, we show a patient-dependent inter- and intraspecific variation for both the immune cell composition and immune checkpoint expression in pleural versus ascites MPM effusions. Nevertheless, PD-1, PD-L1, TIM-3 and LAG-3 are expressed in the majority of effusion samples, thereby identifying TIM-3 and LAG-3 as potential novel targets in MPM. Our data describe two MPM effusion-related factors with clinical value. The percentage of CD4^+^ T cells present in the effusions is significantly correlated with response to chemotherapy, while the percentage of PD-L1^+^ PDPN^+^ tumor cells can be translated as a significant prognostic factor for worse outcome. The results of this study provide more insight in the cellular composition of MPM effusions and support further elaborate research on MPM effusions for the identification of biomarkers and the development of immune checkpoint-targeted immunotherapy.

## SUPPLEMENTARY MATERIALS FIGURES AND TABLES



## Abbreviations

CD: Cluster of differentiation
CTLA-4: Cytotoxic T lymphocyte antigen-4
DC: Dendritic cell
DMSO: Dimethylsulfoxide
FBS: Fetal bovine serum
FDA: Food and drug administration
LAG-3: Lymphocyte activation gene-3
mAb: Monoclonal antibody
MFI: Mean fluorescence intensity
MPM: Malignant pleural mesothelioma
NK: Natural killer
PBS: Phosphate buffered saline
PD-1: Programmed death-1
PD-L1: Programmed death-ligand 1
PDPN: Podoplanin
RECIST: Response evaluation criteria in solid tumors
TIM-3: T cell immunoglobulin mucin-3
TME: Tumor microenvironment
