# Supplementary material for: Abundant expression of TIM-3, LAG-3, PD-1 and PD-L1 as immunotherapy checkpoint targets in effusions of mesothelioma patients
Source: Oncotarget. 2017 Sep 21;8(52):89722–35. doi: 10.18632/oncotarget.21113 (PMC5685704; doi:10.18632/oncotarget.21113)
Supplement: Supplementary file 1 [file oncotarget-08-89722-s001.pdf]

Abundant expression of TIM-3, LAG-3, PD-1 and PD-L1 as immunotherapy checkpoint targets in effusions of mesothelioma patients

SUPPLEMENTARY MATERIALS

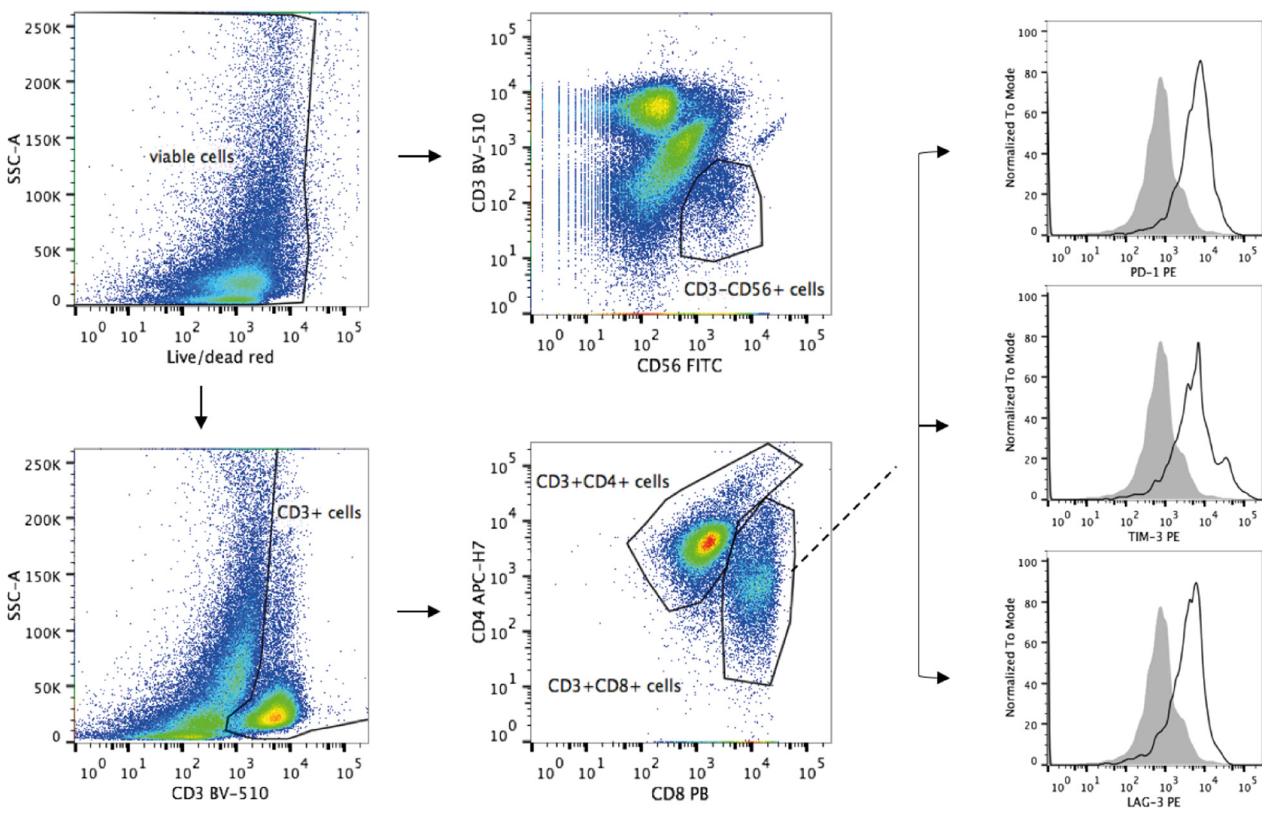

Supplementary Figure 1: Gating strategy to determine immune composition and checkpoint expression. Dead cells were excluded based on the viability staining. Based on CD3 expression in the viable cell population, gates were drawn for NK cells, $CD4^+$ and $CD8^+$ T cells. Expression of PD-1, TIM-3 and LAG-3 is illustrated by the histogram overlays: isotype control (filled grey histogram), specific signal (black line histogram). Representative plots are shown.

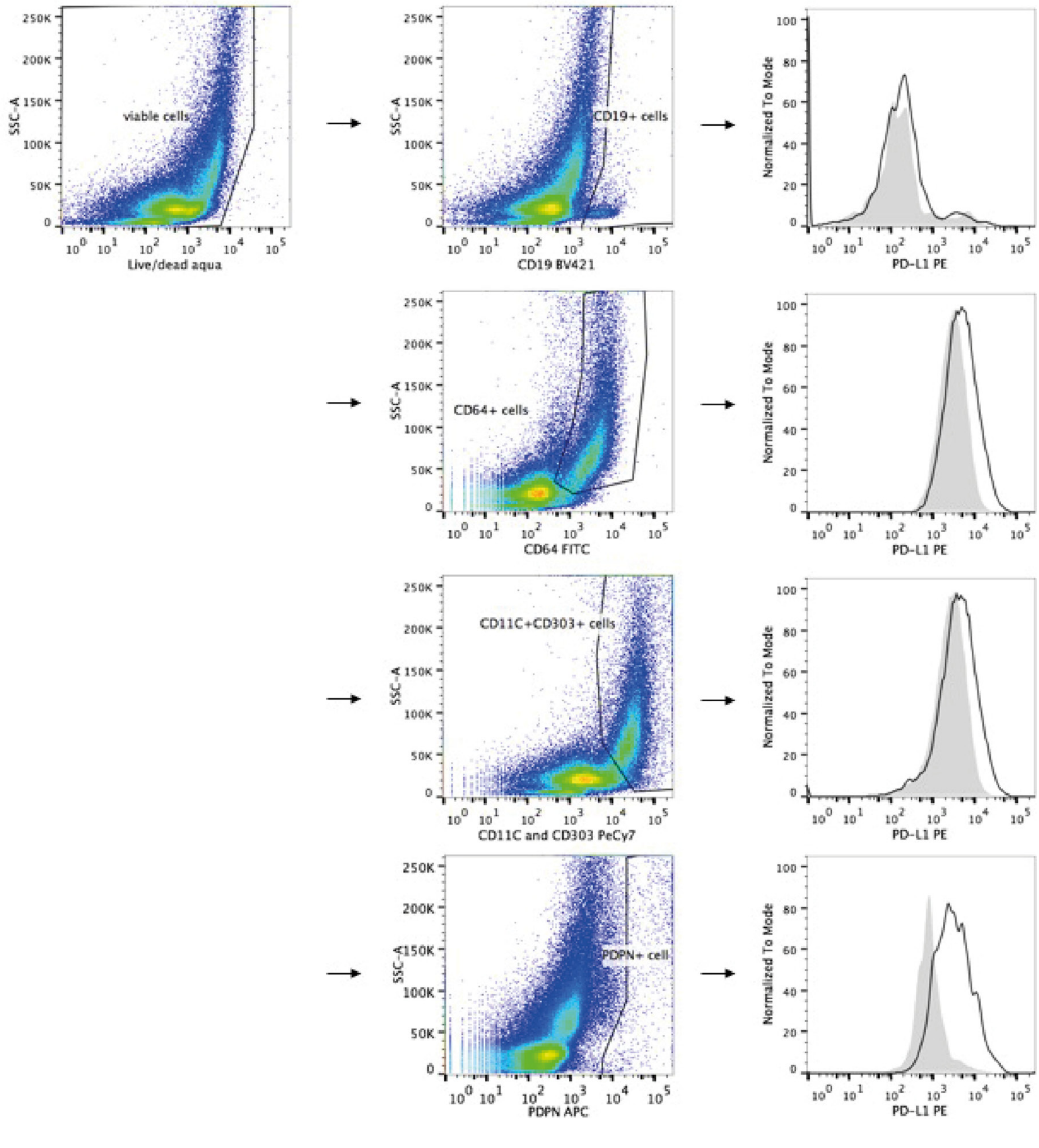

Supplementary Figure 2: Gating strategy to determine immune composition and PD-L1 expression. Dead cells were excluded based on the viability staining. Within the viable cell population gates were drawn for B cells, macrophages, DCs and PDPN+ tumor cells. Expression of PD-L1 is illustrated by the histogram overlays: isotype control (filled grey histogram), specific signal (black line histogram). Representative plots are shown.

Supplementary Table 1: Comparison of 4 patient-matched tissue and fluid samples

SAMPLE	CELL TYPE	IHC			FCM		
		Marker	%	Category	Marker	%	Category
1	PD-L1+ tumor cells	PD-L1+ tumor cells	< 1	0	PD-L1+ PDPN+ cells	0.01	0
	CD4+ lymphocytes	CD4+ lymphocytes in stroma and tumor	10–50	3	Viable CD4+ cells within the CD3+ population	2.99	1
	CD8+ lymphocytes	CD8+ lymphocytes in stroma and tumor	10–50	3	Viable CD8+ cells within the CD3+ population	0.80	0
	Macrophages	CD68+ cells in stroma and tumor	> 50	4	Viable CD64+ cells	0.46	0
	PD-1+ lymphocytes	PD-1+ lymphocytes in stroma and tumor	< 1	0	Sum of PD-1+ CD4/CD8/CD56+ cells	1.21	1
	TIM-3+ lymphocytes	TIM-3+ lymphocytes in stroma and tumor	< 1	0	Sum of TIM-3 CD4/CD8/CD56+ cells	0.45	0
	LAG-3+ lymphocytes	LAG-3+ lymphocytes in stroma and tumor	< 1	0	Sum of LAG-3 CD4/CD8/CD56+ cells	0.80	0
2	PD-L1+ tumor cells	PD-L1+ tumor cells	< 1	0	PD-L1+ PDPN+ cells	0.02	0
	CD4+ lymphocytes	CD4+ lymphocytes in stroma and tumor	5–10	2	Viable CD4+ cells within the CD3+ population	31.16	3
	CD8+ lymphocytes	CD8+ lymphocytes in stroma and tumor	> 50	4	Viable CD8+ cells within the CD3+ population	5.84	2
	Macrophages	CD68+ cells in stroma and tumor	> 50	4	Viable CD64+ cells	3.31	1
	PD-1+ lymphocytes	PD-1+ lymphocytes in stroma and tumor	10–50	3	Sum of PD-1+ CD4/CD8/CD56+ cells	13.05	3
	TIM-3+ lymphocytes	TIM-3+ lymphocytes in stroma and tumor	< 1	0	Sum of TIM-3 CD4/CD8/CD56+ cells	11.56	3
	LAG-3+ lymphocytes	LAG-3+ lymphocytes in stroma and tumor	< 1	0	Sum of LAG-3 CD4/CD8/CD56+ cells	11.44	3
3	PD-L1+ tumor cells	PD-L1+ tumor cells	< 1	0	PD-L1+ PDPN+ cells	0.01	0
	CD4+ lymphocytes	CD4+ lymphocytes in stroma and tumor	10–50	3	Viable CD4+ cells within the CD3+ population	4.45	1
	CD8+ lymphocytes	CD8+ lymphocytes in stroma and tumor	> 50	4	Viable CD8+ cells within the CD3+ population	0.91	0
	Macrophages	CD68+ cells in stroma and tumor	> 50	4	Viable CD64+ cells	0.46	0
	PD-1+ lymphocytes	PD-1+ lymphocytes in stroma and tumor	< 1	0	Sum of PD-1+ CD4/CD8/CD56+ cells	1.24	1
	TIM-3+ lymphocytes	TIM-3+ lymphocytes in stroma and tumor	< 1	0	Sum of TIM-3 CD4/CD8/CD56+ cells	0.60	0
	LAG-3+ lymphocytes	LAG-3+ lymphocytes in stroma and tumor	< 1	0	Sum of LAG-3 CD4/CD8/CD56+ cells	0.49	0
4	PD-L1+ tumor cells	PD-L1+ tumor cells	< 1	0	PD-L1+ PDPN+ cells	0.03	0
	CD4+ lymphocytes	CD4+ lymphocytes in stroma and tumor	< 1	0	Viable CD4+ cells within the CD3+ population	33.83	3
	CD8+ lymphocytes	CD8+ lymphocytes in stroma and tumor	> 50	4	Viable CD8+ cells within the CD3+ population	2.45	1
	Macrophages	CD68+ cells in stroma and tumor	10–50	3	Viable CD64+ cells	15.7	3
	PD-1+ lymphocytes	PD-1+ lymphocytes in stroma and tumor	< 1	0	Sum of PD-1+ CD4/CD8/CD56+ cells	22.91	3
	TIM-3+ lymphocytes	TIM-3+ lymphocytes in stroma and tumor	< 1	0	Sum of TIM-3 CD4/CD8/CD56+ cells	19.34	3
	LAG-3+ lymphocytes	LAG-3+ lymphocytes in stroma and tumor	< 1	0	Sum of LAG-3 CD4/CD8/CD56+ cells	17.67	3

Identification of tumor cells and lymphocytes using IHC was based on morphological analysis. In order to compare the immune composition and checkpoint expression in tissue and fluid samples different scoring categories were used: 0 (≤ 1%), 1 (1–5%), 2 (5–10%), 3 (10–50%), 4 (> 50%). FCM, flow cytometry; IHC, immunohistochemistry; PDPN, podoplanin.
